# Retrospective analysis in oculocutaneous albinism patients for the 2.7 kb deletion in the *OCA2* gene revealed a co-segregation of the controversial variant, p.R305W

**DOI:** 10.1186/s13578-017-0149-3

**Published:** 2017-04-26

**Authors:** Jackson Gao, Leera D’Souza, Keith Wetherby, Christian Antolik, Melissa Reeves, David R. Adams, Santa Tumminia, Xinjing Wang

**Affiliations:** 1 DNA Diagnostic Laboratory, Ophthalmic Genetics and Visual Function Branch, National Eye Institute, National Institutes of Health, 10 Center Drive, MSC 1860, Bethesda, 20892 Maryland USA; 2 National Human Genome Research Institute, Bethesda, Maryland USA; 3 National Eye Institute, National Institutes of Health, Bethesda, Maryland USA

**Keywords:** Albinism, *TYR* gene, *OCA2* gene, p.R305W, Deletion, Linkage disequilibrium

## Abstract

**Background:**

Oculocutaneous albinism (OCA) is an autosomal recessive disorder. A significant portion of OCA patients has been found with a single pathogenic variant either in the *TYR* or the *OCA2* gene. Diagnostic sequencing of the *TYR* and *OCA2* genes is routinely used for molecular diagnosis of OCA subtypes. To study the possibility that genomic abnormalities with single or multiple exon involvement may account for a portion of the potential missing pathogenic variants (the second), we retrospectively analyzed the *TYR* gene by long range PCR and analyzed the target 2.7 kb deletion in the *OCA2* gene spanning exon 7 in OCA patients with a single pathogenic variant in the target genes.

**Results:**

In the 108 patients analyzed, we found that one patient was heterozygous for the 2.7 kb *OCA2* gene deletion and this patient was positive with one pathogenic variant and one possibly pathogenic variant [c.1103C>T (p.Ala368Val) + c.913C>T (p.R305W)]. Further analysis of maternal DNA, and two additional OCA DNA homozygous for the 2.7 kb deletion, revealed that the phenotypically normal mother is heterozygous of the 2.7 kb deletion and homozygous of the p.R305W. The two previously reported patients with homozygous of the 2.7 kb deletion are also homozygous of p.R305W.

**Conclusions:**

Among the reported pathogenic variants, the pathogenicity of the p.R305W has been discussed intensively in literature. Our results indicate that p.R305W is unlikely a pathogenic variant. The possibility of linkage disequilibrium between p.R305W with the 2.7 kb deletion in *OCA2* gene is also suggested.

**Electronic supplementary material:**

The online version of this article (doi:10.1186/s13578-017-0149-3) contains supplementary material, which is available to authorized users.

## Background

Albinism is a disease caused by a lack of or reduction in the production of melanin pigment. There are many genes involved in the complex production of melanin, known as melanogenesis. Albinism affects about 1 in 17,000 individuals worldwide, with varied subtypes and prevalence among different ethnic populations [1]. Oculocutaneous albinism (OCA) is associated with a hypo-pigmented state of melanin in skin, hair, and eyes. In addition, OCA may manifest with eye symptoms such as photophobia, strabismus, nystagmus, and reduced visual acuity. Nonsyndromic OCA describes the disorders in which the manifestations are apparently limited to skin and eye functions. Nonsyndromic OCA is an autosomal recessive condition with genetic heterogeneity. Six genes and one additional locus have been identified as responsible for seven nonsyndromic OCA subtypes [2]. They are OCA1 (*TYR* gene), OCA2 (*OCA2* gene), OCA3 (*TYRP1* gene), OCA4 (*SLC45A2* gene), OCA6 (*SLC24A5* gene), and OCA7 (*C10orf11* gene). OCA5 was identified by linkage analysis to a genomic locus at 4q24 [3]. OCA1 is the most common subtype found in Caucasians and accounts for approximately 50% of cases worldwide [4, 5]. OCA-2 accounts for 30% of cases worldwide and is most prevalent among African and African-American populations. OCA2 is estimated to affect one in 10,000 individuals of African background, while it affects 1 in 36,000 individuals of Caucasian background [6].

The *OCA2* gene (also known as Pink-Eyed Dilution, *P* gene) codes for a membrane protein localized at melanosomes in the melanocytes. Its function may involve transport of tyrosine and some other small molecules [7]. The OCA2 protein may also play a role in stabilizing some melanosomal proteins such as tyrosinase and initiating melanin synthesis in melanocytes. Mutations in the *OCA2* gene (cause the OCA2 phenotype (MIM 203200). The *OCA2* gene consists of 24 exons (23 coding), spanning almost 345 kb of genomic DNA in the region of 15q11.2-q12. The *OCA2* genomic location is also highly polymorphic.

In this study, we evaluated 108 clinically diagnosed OCA patients by sequencing their *TYR* and *OCA2* genes. Then, we retrospectively analyzed the previously reported 2.7 kb *OCA2* gene deletion using long-range PCR. We also confirmed the co-segregation of the deletion and the known controversial variant, p.R305W. We suggested that the p.R305W was the more ancestral allele and the 2.7 kb deletion was introduced into this allele more recently. This association will have a clinical impact on the pathogenicity assessment of the p.R305W variant and genetic consultation.

## Results

In our past clinical genetic tests, OCA patients were sequenced for pathogenic variants in the *TYR* gene at the Molecular Diagnostics Laboratory in the University of Minnesota Medical Center, Fairview (Table 1). In the 68 OCA patients tested for the *TYR* gene mutations, single pathogenic variant was found in 23 patients and 2 pathogenic variants were found in 45 patients. Long range PCR was performed on these patients with a series of overlapped primer sets and with OCA patients with two pathogenic variants including one equivocal mutation of the single mutation samples as PCR controls (Additional file 1: Table S1). No abnormal PCR fragment was identified (data not shown). *OCA2* gene sequencing test was developed later and used as a reflex clinical test for the patients without two pathogenic variants in the *TYR* gene sequencing at the National Eye Institute (Table 2). For the patients without pathogenic variant or with single pathogenic variant or variants with unknown significance in either *TYR* gene or *OCA2* gene, we analyzed the *OCA2* gene 2.7 kb exon 7 deletion. We found that none of these patients had the common exon 7 deletion. We then extended our exon 7 deletion analysis to the rest of the samples in the Tables 1 and 2. We discovered that one patient (NE27) was indeed heterozygous for the exon 7 deletion (Fig. 2). Interestingly, this patient NE27 was also positive for one known *OCA2* gene pathogenic variant, c.1103C>T, p.Ala368Val [8], and one variant with uncertain significance but likely pathogenic, c.913C>T, p.R305W, as we interpreted based on our previous analysis and literature search. Moreover, the patient NE27’s mother was phenotypically normal. We found that this mother was homozygous for the p.R305W variant (data not shown) and heterozygous for the exon 7 deletion (Fig. 2), suggesting the variant of p.R305W is likely to be a benign variant. In addition, the ExAC reported 383 homozygous of p.R305W in 119616 alleles worldwide (ExAC, 3/12/2017). This is strongly support the variant is polymorphism not a pathogenic mutation.

**Table 1:** *TYR* gene genotypes of OCA patients tested at University of Minnesota

Patient ID	Allele 1	Allele 2
MN1	c.1118C>A, p.T373K	
MN2	c.1118C>A, p.T373K	
MN3	c.1118C>A, p.T373K	
MN4	c.665T>C, p.I222T	
MN5	c.1118C>A, p.T373K	
MN6	c.446A>G, p.Y149C	
MN7	c.242C>T, p.P81L	
MN8	c.140G>A, p.G47D	
MN9	c.346C>T, p.R116*	
MN10	c.832C>T, p.R278*	
MN11	c.650G>A, p.R217Q	
MN12	c.286dupA, p.M96Nfs*73	
MN13	c.71G>A, p.C24Y	
MN14	c.1037-7T>A, IVS2-7	
MN15^a^	c.820-2A>G, IVS1-2	
MN16	c.572delG, p.G191Dfs*35	
MN17	c.1467dupT, p.A490Cfs*20	
MN18	c.242C>T, p.P81L	
MN19	c.1118C>A, p.T373K	
MN20	c.823G>T, p.V275F	
MN21	c.1366+4 A>G, IVS4+4	
MN22	c.1366+4 A>G, IVS4+4	
MN23	c.242C>T, p.P81L	c.649C>T, p.R217W
MN24	c.929dupC, p.R311Kfs*7	c.929dupC, p.R311Kfs*7
MN25	c.140G>A, p.G47D	c.140G>A, p.G47D
MN26^a^	c.820-7T>A, IVS1-7	c.1090A>C, p.N364H
MN27^a^	c.732_733delTG, p.Cys244*	c.823G>T, p.V275F
MN28	c.1111A>T, p.N371Y	c.1118C>A, p.T373K
MN29^a^	c.1184+1 G>A, IVS3+1	c.1184+1 G>A, IVS3+1
MN30	c.140G>A, p.G47D	c.140G>A, p.G47D
MN31	c.1118C>A, p.T373K	c.1467dupT, p.A490Cfs*20
MN32	c.661G>A, p.E221K	c.1118C>A, p.T373K
MN33	c.1A>G, p.M1?	c.1217C>T, p.P406L
MN34	c.242C>T, p.P81L	c.1204C>T, p.R402*
MN35	c.896G>A, p.R299H	c.1199G>T, p.W400L
MN36	c.242C>T, p.P81L	c.1036+2T>G, IVS2+2
MN37	c.1217C>T, p.R406L	c.1392dup, p.Lys465* (novel)
MN38	c.832C>T, p.R278*	c.1264C>T, p.R422W
MN39^a^	c.32G>A, p.W11*	c.895C>A, p.R299S
MN40	c.606T>G, p.H202Q	c.1036+2T>G, IVS2+2
MN41	c.242C>T, p.P81L	c.1118C>A, p.T373K
MN42^a^	c.572delG, p.G191Dfs*35	c.1150C>G, p.P384A
MN43	c.1037-7T>A, IVS2-7	c.1037-1G>A, IVS2-1
MN44	c.242C>T, p.P81L	c.1204C>T, p.R402*
MN45	c.1118C>A, p.T373K	c.1147G>A, p.D383N
MN46	c.242C>T, p.P81L	c.1037-7T>A, IVS2-7
MN47	c.242C>T, p.P81L	c.1217C>T, p.P406L
MN48^a^	c.149C>T, p.S50L	c.892C>T, p.R298W
MN49	c.678 680delAGG, p.G227del	c.1118C>A, p.T373K
MN50	c.613C>A, p.P205T	c.823G>T, p.V275F
MN51	c.650G>A, p.R217Q	c.1118C>A, p.T373K
MN52	c.71G>A, p.C24Y	c.758G>A, p.G253E
MN53	c.242C>T, p.P81L	c.1111A>T, p.N371Y
MN54	c.1147G>A, p.D383N	c.1309G>A, p.D437N
MN55	c.823G>T, p.V275F	c.1118C>A, p.T373K
MN56^a^	c.895C>T, R299C	c.1199G>T, W400L
MN57	c.1265G>A, p.R422Q	c.1467dupT, p.A490Cfs*20
MN58	c.1118C>A, p.T373K	c.1209G>T, p.R403S
MN59	c.229C>T, p.R77W	c.572delG, p.G191Dfs*35
MN60	c.140G>A, p.G47D	c.650G>A, p.R217Q
MN61	c.896G>A, p.R299H	c.896G>A, p.R299H
MN62	c.649C>T, p.R217W	c.1118C>A, p.T373K
MN63	c.230G>A, p.R77Q	c.230G>A, p.R77Q
MN64	c.1118C>A, p.T373K	c.1118C>A, p.T373K
MN65	c.1146C>A, p.N382K	c.649delC, p.R217Gfs*9
MN66	c.230G>A, p.R77Q	c.230G>A, p.R77Q
MN67	c.642 644delCTT, p.F214del	c.930_931insG, p.R311Efs*7 (novel)
MN68	c.650G>A, p.R217Q	c.823G>T, p.V275F

Asterisk represents termination codon

^a^The pathogenic alleles in these patients have been reported previously [7]

**Table 2:** The genotypes of TYR and/or OCA2 genes in OCA patients in reflex testing at NEI

Patient ID^a^	TYR		OCA2	
	Allele 1	Allele 2	Allele 1	Allele 2
NE1	c.1118C>A (p.Thr373Lys)			
NE2	Negative		c.1441G>A (p.Ala481Thr)	c.2228C>T (p.Pro743Leu)
NE3	Negative		c.2228C>T (p.Pro743Leu)	
NE4	c.346C>T (p.Arg116^a^)	c.896G>A (p.Arg299His)	Not tested	
NE5^b^	Negative		Negative	
NE6	c.816G>C (p.Trp272Cys)	c.1118C>A (p.Thr373Lys)	Not tested	
NE7^b^	Negative		Negative	
NE8	Negative		c.1327G>A (p.Val443Ile)	c.1842+1G>T (IVS17+1G>T)
NE9	c.1217T>C (p.Pro406Leu)	c.1291C>A (p.Pro431Thr)	Not tested	
NE10	c.1217T>C (p.Pro406Leu)	c.1291C>A (p.Pro431Thr)	Not tested	
NE11	c.238T>C (p.Trp80Arg)	c.1037-7T>A (IVS2-7T>A)	Not tested	
NE12	Negative		c.1465A>G (p.Asn489Asp)	c.2310T>G (p.Tyr770^a^)
NE13	Negative		c.1103C>T (p.Ala368Val)	
NE14	c.929dupC (p.Arg311Lysfs^a^7)	c.929dupC (p.Arg311Lysfs^a^7)	Not tested	
NE15			c.157delA (p.Arg53Glyfs^a^49)	
NE16	c.1118C>A (p.Thr373Lys)		Negative	
NE17	Negative		c.1256G>A (p.Arg419Gln) VUS	
NE18	Negative		c.632C>T (p.Pro211Leu)	c.1240-1G>C (IVS12-1G>C)
NE19	Negative		c.1256G>A (p.Arg419Gln) VUS	
NE20	Negative		c.1372G>A (p.Val443Ile)	
NE21	c.1118C>A (p.Thr373Lys)		c.913C>T (p.Arg305Trp) VUS	
NE22	c.242C>T (p.Pro81Leu)		c.913C>T (p.Arg305Trp) VUS	
NE23	Negative			
NE24	c.1037-1G>A (IVS2-1G>A)	c.1118C>A (p.Thr373Lys)	Not tested	
NE25	c.61C>T (p.Pro21Ser)	c.1037-7T>A (IVS2-7T>A)	Not tested	
NE26	c.1037-7T>A (IVS2-7T>A)	c.347G>T (p.Arg116Leu)	Not tested	
NE27	Negative		c.1103C>T (p.Ala368Val)	c.913C>T (p.R305W)
NE28	Negative			
NE29	c.1118C>A (p.Thr373Lys)		Not tested	
NE30	c.229_230insAGG (p.Arg77delinsGlnGly)		c.913C>T (p.Arg305Trp) VUS	
NE31	c.1366+4A>G (IVS4+4A>G)		Not tested	
NE32	c.346C>T (p.Arg116^a^)	c.929dupC (p.Arg311Lysfs^a^7)	Not tested	
NE33	Negative		c.1256G>A (p.Arg419Gln) VUS	
NE34	c.242C>T (p.Pro81Leu)		Not tested	
NE35	c.1037-7T>A (IVS2-7T>A)		Not tested	
NE36	Negative		c.1465A>G (p.Asn489Asp)	c.2228C>T (p.Pro743Leu)
NE37	Negative		c.1327G>A (p.Val443Ile)	c.1327G>A (p.Val443Ile)
NE38	Negative			
NE39	c.242C>T (p.Pro81Leu)	c.1217C>T (p.Pro406Leu)		

^a^NE13-NE25, NE27-NE29 have been submitte to BRICS for research (Additional file 2: Table S2)

^b^These two samples were found positive for pathogenic alleles in other OCA related genes later

**Fig. 1:**
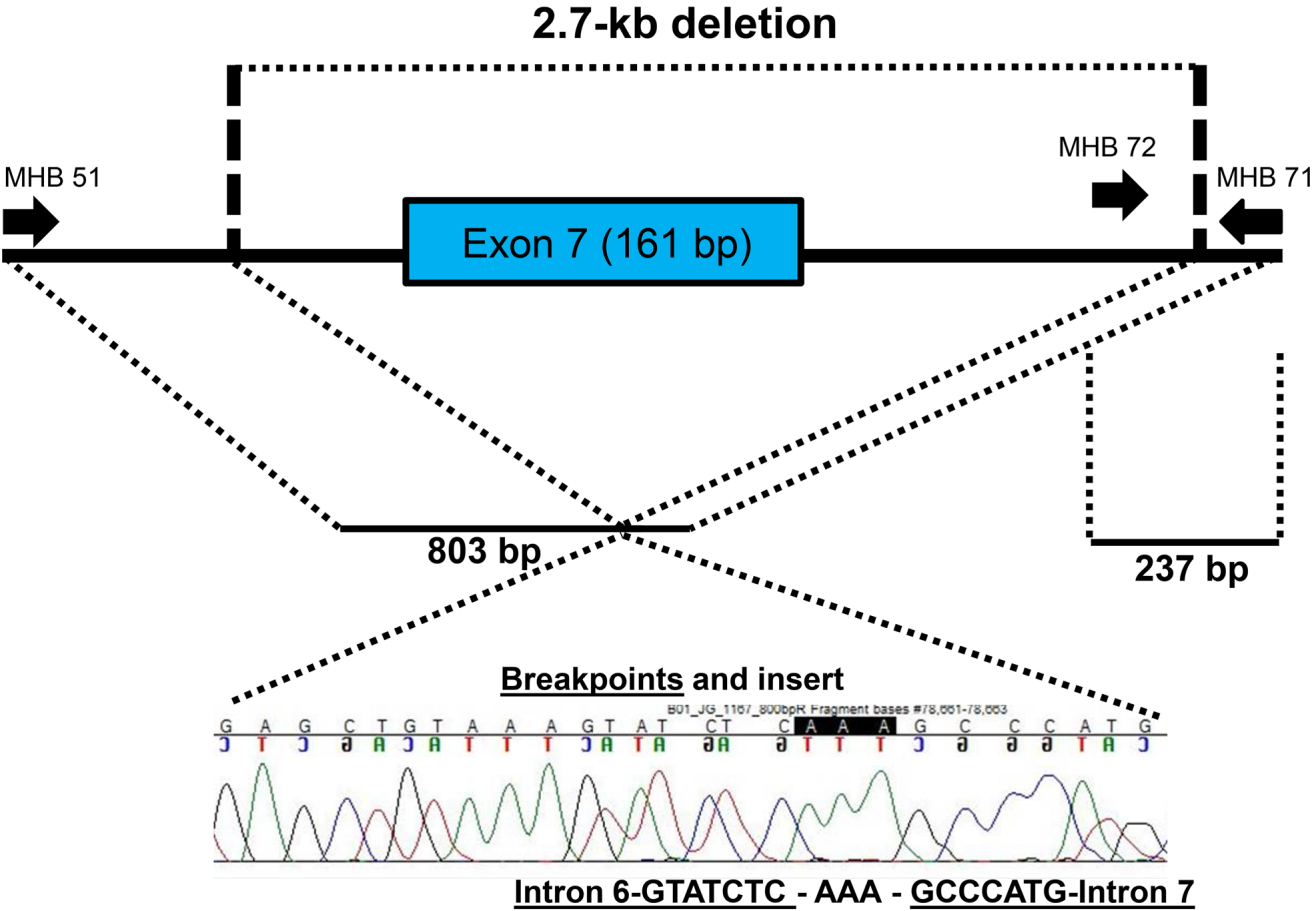
Analysis of the 2.7 kb deletion through PCR and Sanger sequencing. Genomic region around the exon 7 of the *OCA2* gene showed the primer locations, exon 7 and identified deleted region. The expected PCR products were illustrated. The breakpoints of the deletion was confirmed in the Sanger sequencing

We sequenced the 2.7 kb deletion junction fragment isolated from patient NE27 and determined the exact genomic abnormality for this deletion (Fig. 1) as reported previously [7]. Our study identified the deletion of 2958 bases at g.28,017,720_28,020,677 (GRCh38) and followed by replacement with triple A (c.646+1823_c.807+678delinsAAA, p.?), which deleted the exon 7 of *OCA2* gene, leading to an exon skip and potential frameshift (because the exon 7 contains 161 bp which is not in-frame if deleted). This deletion generated an 802 bp PCR fragment by the MHR51F and MHR72R primers. We further analyzed two previously reported homozygotes of the 2.7 kb deletion for the p.R305W variant [7] and found that they were all homozygous for p.R305W too. One additional patient who was heterozygous for the deletion was also heterozygous for the p.R305W variant. It clearly indicated the phase that the 2.7 kb deletion spanning the exon 7 of *OCA2* gene was indeed in cis with the p.R305W variant in the exon 9 of *OCA2* gene.

## Discussion

In our study, we sequenced DNA samples from 108 OCA patients for *TYR* or *OCA2* gene pathogenic variants. Our results consistently supported the published allele distributions [4]. We also performed long range PCR to search for potential large genomic rearrangements such as deletion or duplication in the *TYR* locus and for the 2.7 kB deletion in *OCA2* gene. We did not identify any abnormality at the *TYR* intronic regions using the long range PCR methodology, suggesting that other mechanisms, such as splicing error variants, might be involved. We cannot rule out that this approach was not sensitive enough for smaller sizes of deletions/insertion or even larger massive genomic rearrangements [10]. In addition, we also noticed high frequent of the Alu repetitive sequences in the *TYR* intronic regions.

**Fig. 2:**
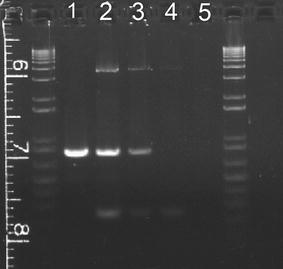
PCR determination of genotypes of the 2.7 kb deletion in OCA patients. Invitrogen 1 Kb Plus DNA ladder were on both sides. *Lane 1* is from a patient who was homozygous of the 2.7 kb deletion [7]. *Lane 2* was NE27. *Lane 3* was the mother of NE27. *Lane 4* was patient NE28. *Lane 5* was no DNA control

The three primer PCR assay system that we adapted from Durham et al. [9] enabled us to determine the genotype of the individuals in a single multiplex PCR with an internal wild type intron sequence control. Observation of the 802 bp deletion-specific junction fragment, the 3.5 kb fragment for the intact genomic region, and the 237 bp fragment spanning one of the breaking points in *OCA2* heterozygotes provided a convenient carrier status confirmation analysis for routine clinical diagnostics (Fig. 2).

In a comparison of gender, ethnic background, genotypes from sequencing results and the deletion status in five samples that were positive for the p.R305W variant (Table 3), besides the patient NE27, we found that the *OCA2* gene in patient NE12 contains two heterozygous pathogenic variants: an A–G change at nucleotide 1465 (c.1465A>G) in exon 14, and a T–G change at nucleotide 2310 (c.2310T>G) in exon 22. These pathogenic variants change the amino acid Asparagine (N) to aspartic acid (D) at residue 489 (p.N489D) and amino acid Tyrosine (Y) to a termination codon (*) at residue 770 (p.Y770*), respectively, in the P protein. The pathogenic variant p.N489D has been identified in unrelated patients diagnosed with Oculocutaneous Albinism Type 2. The pathogenic variant p.Y770* has not been identified in patients diagnosed with Oculocutaneous Albinism Type 2 to our knowledge. However, this nonsense variant is predicted to cause a premature termination in translation and lead to protein product truncation and expected to be pathogenic. The p.R305W was also present in this patient with unknown phase. In three other patients (NE21, NE22, NE30), it was interesting to note that they were all positive for a single pathogenic variant in the *TYR* gene. It was more likely that there would be an unknown secondary pathogenic variant at their *TYR* locus on the other chromosome.

**Table 3:** Comparison of OCA patients bearing the p.R305W variant

IDs	Gender	Ethnic background	*TYR*		*OCA2*	
			Allele 1	Allele 2	Allele 1	Allele2
NE27	Male	American Indian or Alaska Native, Black or African American, White			c.1103C>T (p.Ala368Val)	c.913C>T (p.Arg305Trp) VUS 2.7 kb Exon7 deletion
NE12	Female	White			c.1465A>G (p.Asn489Asp) c.2310T>G (p.Tyr770*) Phase unknown: c.913C>T (p.Arg305Trp) VUS	
NE21	Male	White	c.1118C>A (p.Thr373Lys)		c.913C>T p.Arg305Trp) VUS	
NE22	Female	White	c.242C>T (p.Pro81Leu)		c.913C>T (p.Arg305Trp) VUS	
NE30	Male	Asian	c.229_230insAGG (p.Arg77delinsGlnGly)		c.913C>T (p.Arg305Trp) VUS	

Asterisk represents termination codon

The *OCA2* (*P*) gene consists of 24 exons (23 coding), spanning almost 345 kb of genomic DNA in the region of 15q11.2-q12. The 2.7 kb deletion was expected to result in a frameshift in the first luminal loop, predicting a truncation of the polypeptide and a non-functional gene product. Regarding the protein structure and predicted function, the p.R305 locates in the first luminal loop. Kamaraj and Purohit provided a computational model to depict the functional consequence of p.R305W change [2]. It was predicted that the p.R305W leads to more flexible in conformation, which might alter the catalytic function in melanin biosynthesis. We propose that such functional alteration is not sufficient to lead to OCA. Therefore, homozygous genotype itself would not present OCA and it is demonstrated by unaffected individuals in the population. But in a subpopulation of p.R305W, co-segregated 2.7 kb exon 7 deletion was the real disease-causing mutation. Our previously identified exon 7 deletion positive samples in homozygous and heterozygous status were consistently associated with the p.R305W variant. It suggests that the p.R305W variant was more likely an associated variant as a more ancient allele in linkage disequilibrium. The pathogenicity of the p.R305W has been discussed intensively in literature [2, 3, 11, 12]. Until this study, we were still interpreting that the p.R305W was a VUS with likely pathogenic. Based on the analysis on allele origination, our results are not consistent with the p.R305W variant functioning as a pathogenic variant in an autosomal recessive inheritance pattern. We speculate that the dispute for the pathogenicity of p.R305W was due to lack of analysis on this exon 7 related deletion in the past, which presented as disease associated marker. It should be brought to an attention for our clinical molecular diagnosis on OCA2. More attention should be given to the patient ethnic background and the possibility of a 2.7 kb exon 7 deletion associated with the p.R305W variant in sequencing tests. The p.R305W variant itself is likely benign variant in cis with the exon 7 deletion and likely misleading for OCA accurate molecular diagnosis.

## Conclusion

In summary, we herein evaluated 108 clinically diagnosed OCA patients by sequencing their *TYR* and *OCA2* genes, and alkalized the 2.7 kb *OCA2* gene deletion using long-range PCR. We confirmed the co-segregation of the deletion and the known controversial variant, p.R305W. Our results suggest that the p.R305W is the more ancestral allele and the 2.7 kb deletion is introduced into this allele more recently. These results provided evidence that do not support a pathogenic classification for the p.R305W variant. This association will have a clinical impact genetic consultation for OCA patients.

## Methods

Patients: OCA patients were clinically diagnosed in the United States. Peripheral blood was collected from each patient and DNA was extracted in the Molecular Diagnostics Laboratory in the University of Minnesota Medical Center, Fairview in Minneapolis, Minnesota from 2003 to 2008 and in the NEI DNA Diagnostic Lab at the National Eye Institute (NEI) in Bethesda, Maryland from 2009 to 2016. DNA samples were sequenced for *TYR* and *OCA2* gene pathogenic variants in the Molecular Diagnostics Laboratory in the University of Minnesota Medical Center, Fairview or sequenced in the NEI DNA Diagnostic Laboratory at the National Eye Institute (NEI) in Bethesda, Maryland. This study was reviewed and approved by the University of Minnesota University IRB and the Combined Neuroscience Institutional Review Board of the National Institutes of Health. Informed consent was obtained from each participant as adhering to tenets of the Declarations of Helsinki.

Genomic DNA was extracted using the Puregene genomic DNA extraction kit (Qiagen), and quantified with the ND-1000 spectrophotometer (NanoDrop Technologies, Wilmington, DE). Genomic DNA samples from patients were sequenced using protocol described previously [13].

Long range PCR was performed using the LA PCR kit 2.1 from Takara (Clontech Laboratories, Mountain View, CA). PCR primers for the *TYR* gene intron regions were designed using GeneRunner 3.01 software [13] and PCR condition were optimized by annealing temperatures, magnesium concentrations, and DMSO concentrations (Additional file 1: Table S1). PCR primers for the *OCA2* 2.7 kb deletion and PCR conditions were adapted from Durham-Pierre et al. [9]. For this modified PCR protocol, we used a 2 primer set (MHB51F + MHB71R in a 1:1 ratio) or a 3 primer set (MHB51F + MHB72F + MHB71R in a 4:1:2 ratio) combinations (Fig. 1). The PCR condition was modified as: Denaturation at 94 °C for 1 min, followed by 30 cycles of denaturation at 94 °C for 30 s and annealing at 69 °C for 4 min, followed by final elongation at 72 °C for 10 min. All PCR products were analyzed using 0.8 or 2% agarose gel electrophoresis.

## Additional files

**Additional file 1: Table S1.** Primers for TYR intronic regions.

**Additional file 2: Table S2.** Data accessibility.
