# Supplementary material for: Retrospective analysis in oculocutaneous albinism patients for the 2.7 kb deletion in the OCA2 gene revealed a co-segregation of the controversial variant, p.R305W
Source: Cell Biosci. 2017 Apr 26;7:22. doi: 10.1186/s13578-017-0149-3 (PMC5406851; doi:10.1186/s13578-017-0149-3)
Supplement: Supplementary file 1 — Additional file 1: Table S1. Primers for TYR intronic regions. [file 13578_2017_149_MOESM1_ESM.docx]

| **Additional file 1: Table S1. Primers for TYR intronic regions** | |
| --- | --- |
| TYR gene primers | Sequence 5' to 3' |
| TYR-Intron_F1 | GTGTGACATTTGCACAGATGAG |
| TYR-Intron_R1 | GTGCTCACATCCTGTGTGATC |
| TYR-Intron_F2 | CAGAGTCATGGAATGGAGAAG |
| TYR-Intron_R2 | GGATTTGTCATGGAAACCAGGA |
| TYR-Intron_F3 | GGAGTACAACAGCCATCAGTC |
| TYR-Intron_R3 | GTCAGGGTACACCAAACTACTG |
| TYR-Intron_F4 | CCTCACTAATTCTGAGGGAATC |
| TYR-Intron_R4 | CCAGCAGAAGGGAAACACTG |
| TYR-Intron_F5 | GGTCTTCTAGCACTGTCTAGT |
| TYR-Intron_R5 | CATCGATGGGTCTTGACTATC |
| TYR-Intron_F6 | GACTAACAGCAGATCTCTCAG |
| TYR-Intron_R6 | GAAGCTGTGAGTTAAGGATGAC |
| TYR-Intron_F7 | GTAGTCTCAGCTACTCAGTAGG |
| TYR-Intron_R7 | GCTCTCATACCTAGAGATTCAG |
| TYR-Intron_F8 | GAGATCAGTCCAGCTTAATTC |
| TYR-Intron_R8 | GAAGATAGGATCGTTGGCAG |
| TYR-Intron_F9 | GATTTGCTAGTCCACTTACTG |
| TYR-Intron_R9 | GATTGAGCTTAGTGAGGAAGG |
| TYR-Intron_F10 | GTTTACTGGAGTAGCACTTTGC |
| TYR-Intron_R10 | CAATAAAGACCTGCAACTGTAC |
| TYR-Intron_F11 | CTTTCTGGACTTTGCAACTGG |
| TYR-Intron_R11 | CTACTGATAACCCAGTCGATG |
| TYR-Intron_F12 | GCAGACAGCATCTATGTATCG |
| TYR-Intron_R12 | GGCATACAAGAGACATACCTC |
| TYR-Intron_F13 | GCTAGGACTTCCAGTATTATG |
| TYR-Intron_R13 | GAAGTAGAAGTTCAGGGACTTG |
| TYR-Intron_F14 | CTACCAACTTCCCAGCATATCT |
| TYR-Intron_R14 | CTTTCTGGTTTCCAACATCCTC |
| TYR-Intron_F15 | CCTTGCCTGTTCATATGCAGAT |
| TYR-Intron_R15 | GGATAGAAGATCAAACCAGCTAC |
| TYR-Intron_F16 | CAGATGTGCTGTCACCTAAAC |
| TYR-Intron_R16 | GTGGAGATAGACAGTAACTAGC |
| TYR-Intron_F17 | GCCAGTCCATAACTATAGGTTG |
| TYR-Intron_R17 | GCTATAGTCATAGCCCAGATC |
| TYR-Intron_F18 | CAATGCACCCATTGGACATAAC |
| TYR-Intron_R18 | CTTGGCTGAAGTGCTTTCACA |
| TYR-Intron_F19 | GGAGCAATAGTAAGAGAAGCTG |
| TYR-Intron_R19 | GTCTTGAAAAGAGTCTGGGTC |
