# Supplementary material for: Retrospective analysis in oculocutaneous albinism patients for the 2.7 kb deletion in the OCA2 gene revealed a co-segregation of the controversial variant, p.R305W
Source: Cell Biosci. 2017 Apr 26;7:22. doi: 10.1186/s13578-017-0149-3 (PMC5406851; doi:10.1186/s13578-017-0149-3)
Supplement: Supplementary file 2 — Additional file 2: Table S2. Data accessibility. [file 13578_2017_149_MOESM2_ESM.docx]

| Additional file 2: Table S2. Data accessibility | |
| --- | --- |
| ID in this study | BRICS ID* |
| NE13 | NEI_INVNY854BX7 |
| NE14 | NEI_INVKA491DW4 |
| NE15 | NEI_INVUY245GF9 |
| NE16 | NEI_INVDE573KZN |
| NE17 | NEI_INVHN767BAR |
| NE18 | NEI_INVWW292NMN |
| NE19 | NEI_INVWN196CKF |
| NE20 | NEI_INVAP124BY2 |
| NE21 | NEI_INVPP699AUU |
| NE22 | NEI_INVXA676WUP |
| NE23 | NEI_INVDB623LDQ |
| NE24 | NEI_INVGY790DJX |
| NE25 | NEI_INVGR179FJ6 |
| NE27 | NEI_INVTN769XPU |
| NE28 | NEI_INVEW499DHZ |
| NE29 | NEI_INVYV213FBG |
| NE30 | NEI_INVJJ511BLR |
| NE31 | NEI_INVWA923NUY |
| NE32 | NEI_INVTJ856MK7 |
| NE33 | NEI_INVMG402UJ8 |
| NE34 | NEI_INVFF683UVM |
| NE35 | NEI_INVEE355BTV |
| NE36 | NEI_INVFR825YR4 |
| NE37 | NEI_INVPJ047WMH |
| NE38 | NEI_INVDL151XPV |
| NE39 | NEI_INVVA386AMM |
| * These samples are available for research by their BRICS ID | |
